# Adaptive genome duplication affects patterns of molecular evolution in *Saccharomyces cerevisiae*

**DOI:** 10.1371/journal.pgen.1007396

**Published:** 2018-05-25

**Authors:** Kaitlin J. Fisher, Sean W. Buskirk, Ryan C. Vignogna, Daniel A. Marad, Gregory I. Lang

**Affiliations:** Department of Biological Sciences, Lehigh University, Bethlehem, PA, United States of America; University of British Columbia, CANADA

## Abstract

Genome duplications are important evolutionary events that impact the rate and spectrum of beneficial mutations and thus the rate of adaptation. Laboratory evolution experiments initiated with haploid *Saccharomyces cerevisiae* cultures repeatedly experience whole-genome duplication (WGD). We report recurrent genome duplication in 46 haploid yeast populations evolved for 4,000 generations. We find that WGD confers a fitness advantage, and this immediate fitness gain is accompanied by a shift in genomic and phenotypic evolution. The presence of ploidy-enriched targets of selection and structural variants reveals that autodiploids utilize adaptive paths inaccessible to haploids. We find that autodiploids accumulate recessive deleterious mutations, indicating an increased susceptibility for nonadaptive evolution. Finally, we report that WGD results in a reduced adaptation rate, indicating a trade-off between immediate fitness gains and long-term adaptability.

## Author summary

Whole genome duplications—the simultaneous doubling of each chromosome—can have a profound influence on evolution. Evidence of ancient whole genome duplications can be seen in most modern genomes. Experimental evolution, the long-term propagation of organisms under well-controlled laboratory conditions, yields valuable insight into the processes of adaptation and genome evolution. One interesting, and common, outcome of laboratory evolution experiments that start with haploid yeast populations is the emergence of diploid lineages via whole genome duplication. We show that, under our laboratory conditions, whole genome duplication provides a direct fitness benefit, and we identify several consequences of whole genome duplication on adaptation. Following whole-genome duplication, the rate of adaptation slows, the biological targets of selection change, and aneuploidies, copy-number variants and recessive lethal mutations accumulate. By studying the effect of whole genome duplication on adaptation, we can better understand how selection acts on ploidy, a fundamental biological parameter that varies considerably across life.

## Introduction

The natural life cycle of budding yeast alternates between haploid and diploid phases. Both ploidies can be stably propagated asexually through mitotic division. Both theory and experimental work show that haploids adapt faster than diploids, likely due to recessive beneficial mutations [1,2]. Curiously, however, repeated attempts at evolving experimental haploid populations have resulted in recurrent whole genome duplications yielding populations of autodiploids ([3–5], see **Table 1**). Proposed explanations of this phenomenon include artifacts of strain construction [6], unintended mating events [5], and an adaptive advantage of diploidy [3].

**Table 1:** Observations of autodiploidy in experimental studies.

Study	Propagation	Evolution medium	Strain background	Mating-type
Current study	Batch culture, unshaken	YPD	W303	*MAT***a** & *MAT*α
Kosheleva and Desai 2017	Batch culture, unshaken	YPD	Sk1-W303 hybrid	*MAT***a** & *MAT*α
Gorter 2017	Batch culture, shaken	YPD with heavy metals	BY4743	*MAT***a**
Venkataram *et al*. 2016	Batch culture, shaken	Carbon limited glucose	BY4709	*MAT***a**
Voordeckers *et al*. 2015	Turbidostat	6–12% EtOH glucose	S288c derivative	*MAT*α
Hong and Gresham 2014	Chemostat	Nitrogen limited glucose	S288c derivative	*MAT***a**
Oud *et al*. 2013	Anaerobic batch culture in sequential bioreactor	1:1 glucose/galactose	CEN.PK113-7D	*MAT***a**
Gerstein *et al*. 2006	Batch culture, shaken	YPD	SM2185	*MAT***a**

Whole genome duplication (WGD) in asexual haploid populations could provide a fitness advantage in several different ways. Cell size scales with DNA content across many taxa including yeast [7,8,9], and increased cell size may facilitate more rapid metabolism and increased growth rate. Indeed, increased cell volume has been reported in laboratory-evolved microbial populations [10]. Gene expression patterns also vary with ploidy [11], and diploid-specific gene regulation may be optimal. “Ploidy drive” has been used to describe the phenomenon by which ploidy changes in evolving fungi favor restoration of the historical ploidy state [12]. Natural *Saccharomyces cerevisiae* isolates are typically diploid [13] and occasionally polyploid [14]. If most selection has occurred on these higher ploidy states, then gene regulation and cell physiology of diploids should be better optimized relative to haploids.

Despite the recurrence of diploidization events in haploid-founded yeast lineages, the nature of the fitness advantage of diploidy remains unclear. Some studies detect a fitness benefit [6,15], while no advantage is detected in others [4,16]. A survey of the effect of ploidy on growth rate in otherwise isogenic strains indicates that the benefit of ploidy varies across conditions and optimal ploidy states are contingent on environment [17]. In environments where duplication does not confer a direct fitness advantage, it may afford indirect benefits that are then themselves acted upon by selection. Diploidy may transiently protect evolving lineages from purifying selection by masking the effects of deleterious recessive mutations over short time scales. Indeed, 15% of viable single gene deletions in haploids exhibit growth defects in rich media, while 97% of heterozygous gene deletions show no detectable phenotype in the absence of perturbation [18]. This “masking” hypothesis also has experimental support from mutagenesis studies [19], and this effect could be advantageous in populations in which the deleterious mutation rate is sufficiently high.

Autodiploids could invade haploid populations due to increased access to beneficial mutations. Ploidy-dependent mutations are known to arise in experimental evolution [20,21], and a favorable shift in the distribution of fitness effects may follow genome duplication. Structural variants—deletions, amplifications, and translocations—have repeatedly been shown to be adaptive in experimentally evolving yeast populations [22,23]. Diploids have a greater tendency to form copy number variants (CNVs), especially large deletions [24]. Likewise, aneuploidies accumulate at a significantly higher rate in diploids in the absence of selection [25]. If structural variants are more frequent, more variable, and more tolerable in diploids, genome duplication may enable access to novel adaptive paths. Given the repeated observation of displacement of haploids by diploids (**Table 1**), and the absence of clear evidence for instantaneous fitness advantages of isogenic diploidy that is broadly applicable across experiments, it is possible that selection for and maintenance of diploidy is a complex process involving both direct selection on ploidy state and second order selection, or selection for indirect fitness benefits associated with higher ploidy.

Here we show recurrent WGD in 46 haploid-founded populations during 4,000 generations of laboratory evolution in rich media. We track the dynamics of genome duplication across the haploid-founded populations, revealing that autodiploids fix by generation 1,000 in all 46 populations. Competitive fitness assays show that WGD provides a 3.6% fitness benefit in the selective environment. We find that the immediate fitness gain is accompanied by a loss of access to recessive beneficial mutations. As a consequence, the rate of adaptation of autodiploids slows. Sequencing of the evolved genomes indicates that autodiploids have increased access to structural variants and largely utilize a different spectrum of mutations to adapt compared to haploids. Finally, we show that autodiploids are buffered from the effects of recessive deleterious mutations, consistent with an initial benefit to a newly-formed diploid genome and loss of redundancy following WGD.

## Results

### Sequenced genomes indicate early and recurrent fixation of autodiploids

Two clones were sequenced from each of 46 haploid-founded populations after 4,000 generations of evolution, revealing over 5,100 *de novo* mutations distributed uniformly across the genome, representing the largest dataset of mutations identified in *S*. *cerevisiae* experimental evolution to date (**S1 Fig**; **S1 Dataset**). Mutations are normally distributed across clones (one-sample Kolmogorov-Smirnov test, α = 0.05) with a mean of 91 ± 20 (**S2A Fig**). Most mutations in the sequenced clones were called at ~0.5 (implying heterozygosity), a surprising result given that the populations were founded by a haploid ancestor. Recurrent WGD events were suspected given that each clone maintained its ancestral mating-type allele. Further, this hypothesis of WGD was supported by the observation that clones are not heterozygous at the 6 polymorphic sites that differ between the *MAT***a** and *MAT*α strains. Finally, evolved autodiploids are mating competent, pointing to duplication of haploid genotypes.

### Autodiploids are detected early, sweep quickly, and exhibit a fitness advantage

**Fig 1:**
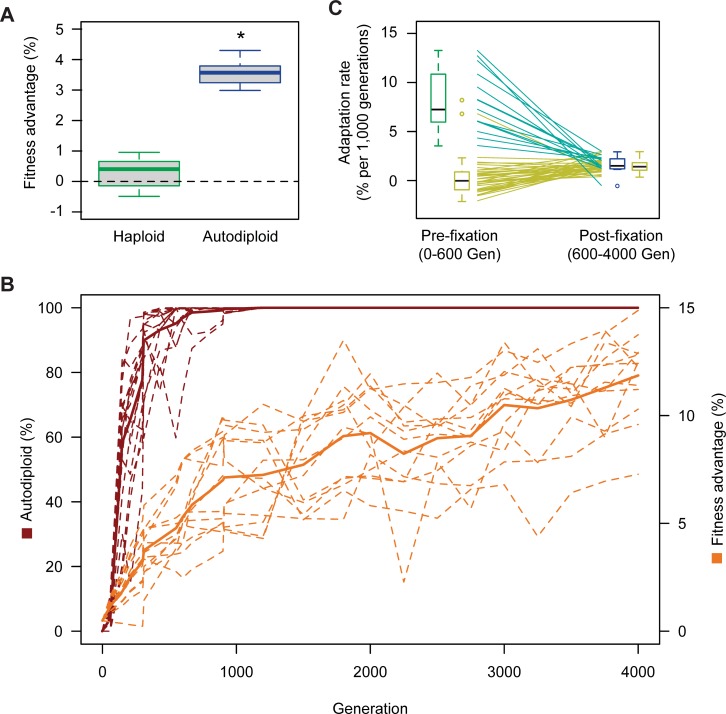
Autodiploids sweep through haploid populations due to a direct fitness advantage. A) *MAT****a****/****a*** diploids have a mean relative fitness advantage of 3.6% when competed against a haploid reference strain. Ten *MAT****a****/****a*** diploids clones were constructed independently. Box plots reflect mean fitness of each clone. Autodiploids and control haploids were competed against the same haploid reference. Asterisk (*) indicates *p*<0.001 (Welch’s t-test, *df* = 18.268) B) Autodiploid frequency (red) and fitness advantage (orange) for focal populations (dashed lines). Solid lines indicate mean autodiploid frequency for 16 populations and mean fitness advantage for 13 populations. C) Haploid-founded populations demonstrate significantly higher rates of adaptation until autodiploids fix in the haploid-founded populations. From that point forward, haploid-founded (autodiploids) and diploid-founded populations adapt at the same rate. Lines indicate paired data points from the same population (teal: haploid-founded, yellow: diploid-founded). For each haploid-founded population, adaptation rate was calculated before and after autodiploid fixation, which occurred on average at generation 600. Adaptation rates for diploid-founded populations (diploid data reported in reference 20) were calculated from Gen 0–600 and Gen 600–4000.

We determined the fitness effect of genome duplication by directly competing *MAT***a**/**a** autodiploids against an otherwise isogenic haploid *MAT***a** reference. To control for possible artifacts of construction, we independently constructed and competed 10 *MAT***a**/**a** diploids. All 10 *MAT***a**/**a** autodiploid reconstructions exhibit a relative fitness advantage significantly higher than a control haploid strain (Welch’s t-test, *t* = 16.28 *df* = 19, *p* < .001). Genome duplication alone in the absence of any other variation provides a mean fitness benefit of 3.6% in these experimental conditions (**Fig 1A**).

To determine the timing of duplication events, we performed time-course DNA content staining on cryoarchived samples for 16 randomly selected populations (8 of each mating-type). Autodiploids arise quickly in all 16 populations, fixing by generation 1,000 in all but 2 populations (**Fig 1B**, **S3 Fig**, **S4 Fig**). Diploids are present at 2% - 11% in 11/16 populations at generation 60, the earliest time point available for assay. Some populations appear to show clonal interference by fit haploids, with autodiploid fractions briefly decreasing between some time points. Aside from such slight variations, patterns of emergence and spread of autodiploids display similar dynamics for all 16 populations examined.

We examined whether the degree of parallelism observed in ploidy dynamics can be attributed to ancestral ploidy polymorphisms present at the onset of the experiment. Four lines of evidence support the independent origin of autodiploidy in this experiment. First, the cultures were initiated from two starting strains (*MAT***a** and *MAT*α). There is no significant difference in autodiploid frequency between mating-types at any generation (**S3 Fig**), meaning if autodiploids did, in fact, arise in both independent inoculating cultures, they would have had to achieve roughly the same frequency, which is highly unlikely. Second, no diploids were detected by DNA content staining in any populations at Generation 0, indicating autodiploids were not present in the inocula above our detection limit of 1%. Third, computational simulations show that low frequency autodiploids are insufficient to explain the recurrent observation of autodiploid fixation events in all 46 replicate populations. Autodiploids with a 3.6% fitness advantage starting at a frequency of 0.01, the highest frequency we modeled, have a probability of fixation in a given population of 0.88 and therefore the chance of fixation in all 46 populations would be 2.5 x 10^−3^ (**S5 Fig**). A fourth line of evidence is the recent reporting of a high rate of autodiploid occurrence in passaged yeast cultures. Harari *et al*. [26] report a rate ploidy transition on the order of 10^−5^ per cell division, which corresponds to hundreds of WGD events generated during each 24-hour growth cycle. Taken together, this argues that, while ancestral autodiploids may have swept in some populations, ancestral ploidy variation is insufficient to explain autodiploid fixation in all 46 populations. Therefore independent, parallel WGD events during the evolution experiment are necessary to explain the recurrent fixation reported here.

### Autodiploids adapt more slowly than haploids

Consistent with previous work [20,27], we find that WGD in haploids provides an immediate fitness gain at the expense of slowing subsequent adaptation. To examine how the shift to diploidy impacted the dynamics of adaptive evolution, we measured population fitness for all populations at ~300-generation intervals. Mean time-course fitness estimates show a change in slope following 1,000 generations. This corresponds roughly to the time that autodiploids have fixed in most focal populations and are high frequency in the remaining populations (**Fig 1B**). We compared the rate of adaptation before and after the fixation of diploids in 13 focal populations for which quality fitness data was available. Because many factors, including epistasis, could explain a change in adaptation rate over time, we used a repeated measures ANOVA to compare the effect of ploidy on adaptation rate using time-course fitness data from diploid-founded populations that were evolved in parallel [20] (**Fig 1C**). The interaction of founding ploidy and generation has a significant effect (*F*(1, 49) = 78.04, *p* < .001, *η*_*p*_^*2*^ = 0.614). Post hoc comparisons using a Bonferroni correction indicate that rates of adaptation are significantly higher in haploid-founded populations than diploids (*p* < .001), and that adaptation rate does not differ once autodiploids fix (*p* = .38). Duplication itself is a significant component of incipient haploid adaptation, however, diploidy alone is unable to account for the range of population fitness values at the time point in which diploids fix, which ranges from 1.9% to 8.0%. Therefore, additional beneficial mutations are needed to explain high gains in fitness in some populations.

### Autodiploid genomes harbor autodiploid specific mutations

**Fig 2:**
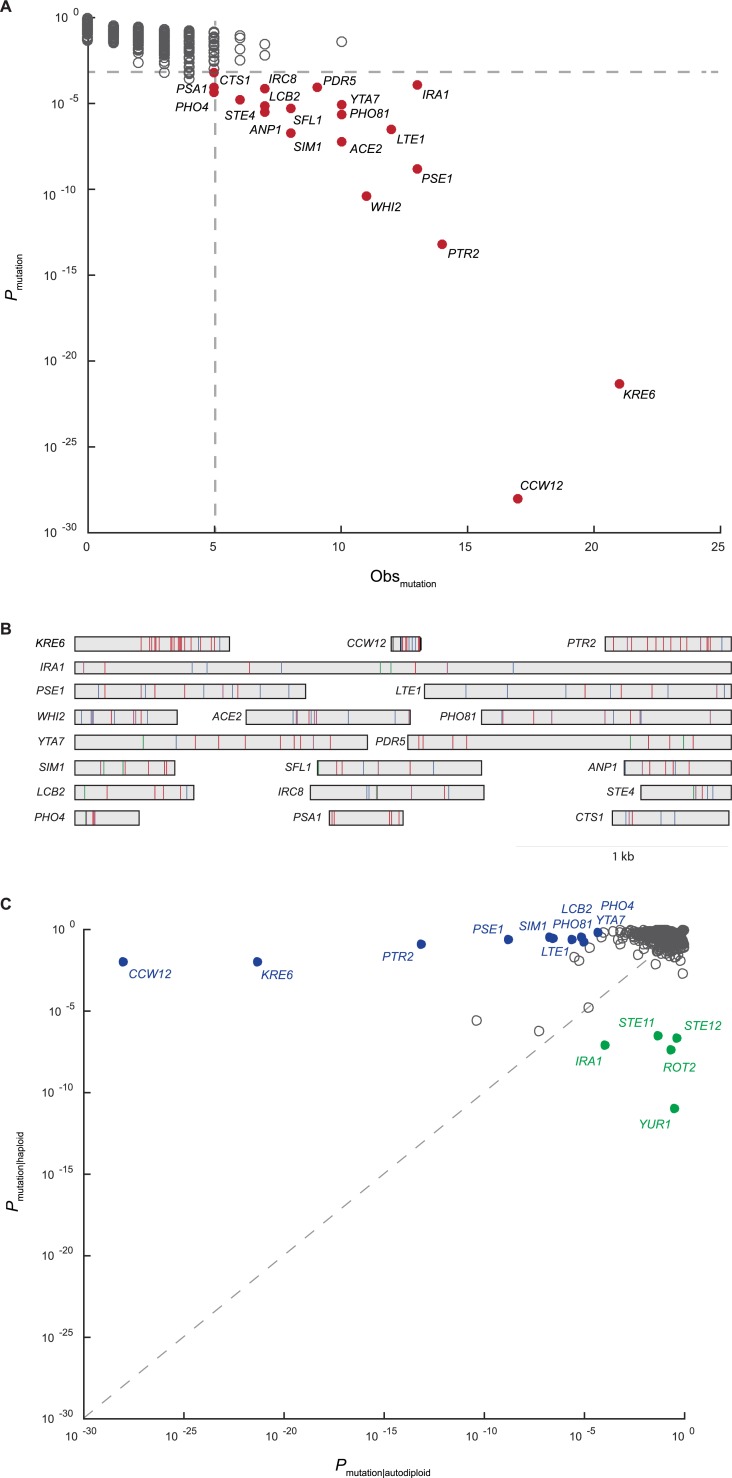
Common targets of selection and ploidy-enriched genes. A) Plotted on the x-axis is the observed number of coding sequence (CDS) mutations in each of the 5800 genes in the S288c reference genome. On the y-axis is the probability that the observed number of CDS mutations in each gene occurred by chance. Common targets of selection (solid red circles) are genes with 5 or more CDS mutations and corresponding probability of less than 0.1%. B) Shown are all 188 mutations across the 20 common targets of selection. Genes are represented as rectangles and labeled by gene name. Mutations are colored by type: frameshift-purple, nonsense-blue, missense-red, synonymous-green, other-black. Both homozygous and heterozygous mutations are shown. C) Plotted is the probability that the observed number of CDS mutations in a gene occurred by chance in haploid populations (haploid data reported in reference 30) versus autodiploid populations. Genes were considered ploidy-enriched if the ratio of probabilities was greater than 10^5^. Haploid-enriched genes are indicated by solid green circles and autodiploid-enriched genes as solid blue circles.

Duplication of a haploid genome affects both cell physiology and the phenotypic consequences of new mutations. Therefore, the selective pressure on a gene may vary depending on ploidy state. To understand how genome evolution is driving adaptation in the autodiploid populations, we utilize a recurrence approach that accounts for both the number of mutations observed in a gene and the expectation that the observed number of mutations of a given gene occurred by chance alone controlling for gene length. The resulting probabilities were used to identify 20 common genic targets of selection (**Fig 2A, S1 Table**). There is a median of four recurrent targets per clone with only one population containing no common target mutations. GO-component term analysis indicates common targets are enriched for genes whose protein products localize to the cell periphery (*p* = 0.001). Cell periphery targets include *CCW12* and *KRE6*, which both appear to be under extremely strong selective pressure when using the probability metric as a proxy for strength of selection. Interestingly, a tRNA gene, tL(GAG)G, was also identified as a common target of selection (**S6 Fig**). This is the first evidence of adaptive tRNA mutations in laboratory yeast evolution.

To better understand the molecular basis of adaptation, we examined the distribution of mutations within each gene (**Fig 2B**). Three broad patterns emerge. First, we observe selection for loss-of-function alleles, e.g. 9 of 11 mutations in *WHI2* are high impact (frameshift or nonsense). Adaptive loss-of-function alleles are common in experimental microbial evolution [6,28,29]. We also observe selection for change-of-function alleles. For example, only missense and synonymous mutations are seen in *PDR5*. Finally, we observe mutations in common targets that cluster within specific domains. This is illustrated by the clustering of mutations in the C-terminus of both *KRE6* (n = 21) and *STE4* (n = 6).

We compared the common targets of selection identified in autodiploid clones to those identified with the same approach in a comparable haploid dataset [30] (**S7 Fig**). We identify several haploid- and autodiploid-enriched targets (**Fig 2C**). Ploidy-enriched targets include genes mutated more often in one ploidy (e.g. *CCW12* and *KRE6* in autodiploids; *YUR1 and ROT2* in haploids) or exclusively in one ploidy (e.g. *PHO81*, *YTA7*, *IRC8* in autodiploids; *STE12* in haploids).

### Loss of heterozygosity hotspots occur on Chromosomes XII and XV

**Fig 3:**
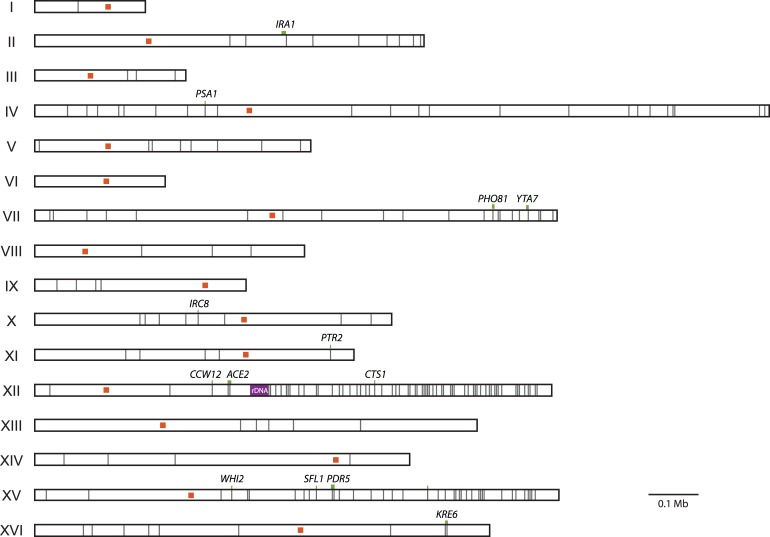
Enrichment of homozygous mutations on the right arms of Chr. XII and Chr. XV. Shown in gray lines are the 256 homozygous mutations detected across the 92 evolved clones. Chromosomes are labeled by Roman numeral. Centromeres are shown as orange squares. Homozygous mutations in common targets of selection are marked by a green line (representing gene length) and labeled by gene name. The ribosomal DNA repeat region of Chr. XII, a known recombination hotspot, is shown in purple.

Though most mutations are heterozygous, clones contain up to 17 homozygous mutations, with an average of 5.4. Homozygous mutations could either represent mutations that arose before duplication events or loss of heterozygosity (LOH) of heterozygous mutations. We find that the homozygous mutations are not distributed randomly throughout the genome; instead, they tend to cluster in particular regions of the genome (**Fig 3**). These clusters, located on the right arms of Chr. XII and Chr. XV, account for 55% of all homozygous mutations. This clustering implies that most homozygous variants result from recombination events. By removing homozygous mutations occurring in these regions from analysis, the average number of homozygous mutations per clone drops to 2.4. This confirms that only a few mutations arose in a haploid background and that most genome evolution occurred post genome duplication.

Mutations in the common targets of selection are observed as both homozygous and heterozygous. Most genes (12/20) are found mutated in both heterozygous and homozygous states across clones, indicating partial or full dominance of fitness effects. Seven genes only ever contain heterozygous mutations (*ANP1*, *LCB2*, *LTE1*, *PHO4*, *SIM1*, *STE4*, *PSE1*). These mutations are candidates for overdominant effects [31]. Finally, only one gene, *CTS1*, is never found mutated in a heterozygous state. A reasonable hypothesis would be that the *cts1* mutations are recessive; however, we have previously identified *cts1* mutations in evolved diploid populations and found it to be close to fully dominant [20]. Instead, the position of *CTS1* on the right arm of Chr. XII, a LOH hotspot, could explain why it is only observed in a homozygous state (**Fig 3**).

### Structural variants are common to autodiploids

**Fig 4:**
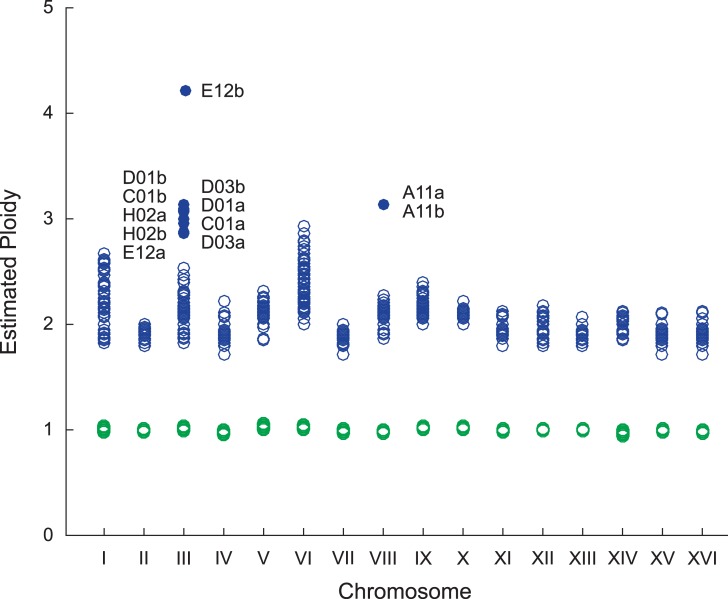
Detection of aneuploidies. For each sequenced sample, coverage across each chromosome was compared to genome-wide coverage. Based on DNA content staining, baseline ploidy was assumed to be 1N for haploids and 2N for autodiploids. Euploidy is indicated by empty circles: haploid—green, autodiploids—blue. Aneuploidies are shown as filled circles and labeled by clone.

In addition to changing the genetic targets of selection, genome duplication permits access to structural variants not accessible to haploid genomes. We analyzed aneuploidies and copy number variants (CNVs) in autodiploid genomes as well as previously sequenced haploid populations [30] (**Figs 4 & S8; S2 and S3 Datasets**). Two types of aneuploidies are observed in autodiploids: trisomy III (which fixes in five populations) and trisomy VIII (which fixes in one) (**Table 2**). CNVs are common in autodiploid genomes. Of the 46 autodiploid populations, CNVs appear in 19 and fix in 14. The 19 independently occurring autodiploid CNVs fall into 10 groups based on genomic position (**Table 2**). Autodiploid CNVs consist of both amplifications (n = 4) and deletions (n = 6). In contrast, no aneuploidies and only two amplifications are detected amongst the 40 haploid populations. These two amplifications are also observed in autodiploids.

**Table 2:** Structural variants in evolved autodiploids.

Chr.	Start (kb)	End (kb)	Length (kb)	Copy Number	Description	Type	Clones*
I	210	225	15	1N	CNV	loss	**B01a**, **B01b**, **E11a**, **E11b**
III	85	85	<10 kb	0N	CNV	loss	**G01a**, **G01b**, **G01c**
III	150	170	20	1N	CNV	loss	**A02a**, **A02b**, **B10a**, **B10b**, **C11a**, **C11b**, **C11c**, F10a
IV^2^	900	1000	100	3N	CNV	gain	**B12a**, **B12b**, C03a, **E12a**, **E12b**
V^3^	450	500	50	1N	CNV	loss	**B11a**, **B11b, F10a**, **F10b**
VIII	525	545	20	1N	CNV	loss	**E11a, E11b**
XIII^3^	190	200	10	1N	CNV	loss	C10a, D10a, E10c, H12a
XIII^2^	190	200	10	3N	CNV	gain	**F02a**, **F02b**
XIV	545	560	15	3N	CNV	gain	**A12a**, **A12b**
XV	900	1100	200	3N	CNV	gain	G02b
III	0	317	317	3N^1^	aneuploidy	gain	**C01a**, **C01b**, **D01a**, **D01b**, **D03a**, **D03b**, **E12a**, **E12b**^1^, **H02a**, **H02b**,
VIII	0	924	924	3N	aneuploidy	gain	**A11a**, **A11b**

* Bolded clones indicate the CNV was found in all clones of the population

^1^ Observed at 4N in one clone

^2^ Also observed in one haploid

^3^ Contains essential genes

### Autodiploids are buffered from deleterious mutations

**Fig 5:**
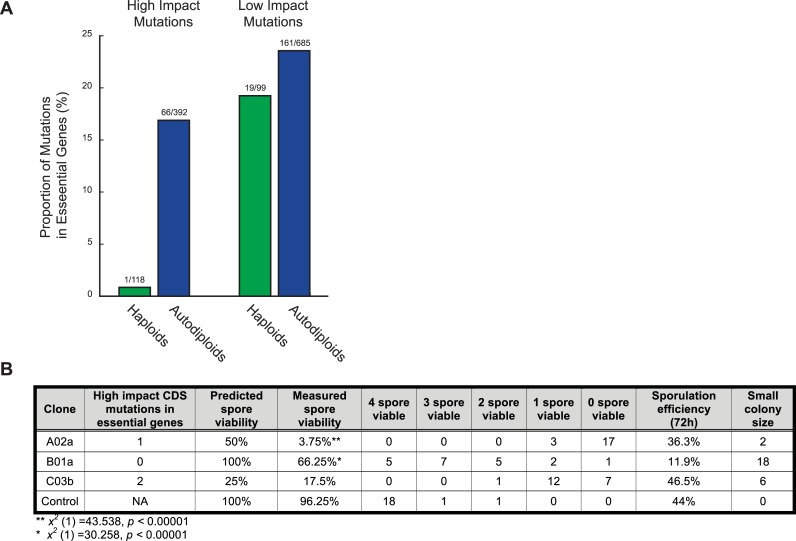
Recessive deleterious and lethal mutations. A) Shown are the proportions of high impact mutations (frameshift, nonsense) and low impact mutations (synonymous, intronic) in essential genes in haploids (green) and autodiploids (blue). Above each bar is the ratio of mutations in essential genes to mutations in all genes. B) Clones from three evolved diploid populations were sporulated and dissected. Spore viability and small colony size reflect recessive lethal and recessive deleterious mutations, respectively.

To determine the extent to which an increase in ploidy buffers diploid lineages against the effects of deleterious mutations, we compared the frequency of mutations in essential genes in autodiploids with those of *MAT***a** haploids described previously [30]. We specifically analyzed frameshift and nonsense mutations that would likely phenocopy the null mutants used to characterize genes as essential. Sixty-three of 66 high impact mutations in essential genes are heterozygous. For the remaining three mutations, zygosity is inconclusive due to low coverage (**S2B Fig**). We find high impact mutations in essential genes to be exceptionally rare in haploids, with only a single case observed (**Fig 5A**). In contrast, autodiploids contain a significantly higher proportion of high impact mutations in essential genes (*x*^*2*^ (1) = 20.32, *p <*0.0001). As expected, the proportion of low impact mutations within essential genes is consistent across ploidies (*x*^*2*^ (1) = 0.909, *p* = 0.339). Essential genes are also present within two of the large deletions observed in autodiploids (**Table 2**).

To experimentally validate that recessive lethal mutations accumulate in autodiploids, we sporulated three *MAT***a**/**a** from three different populations and performed tetrad dissections. Clones A02a, B01a, and C03b were selected because they contain no identifiable aneuploidies that would complicate measures of spore viability. Out of 20 total dissected tetrads (80 total spores) per clone, spore viability ranged from 4% to 66% in evolved autodiploid clones (**Fig 5B**). Further, a substantial fraction of germinated spores developed morphologically small colony sizes relative to controls. We compared observed spore viability to expected viability based on the number of high impact mutations in genes annotated as essential. The only clone for which we observed four-spore viable tetrads, B01a, is also the only clone with no predicted recessive lethal mutations. Nonetheless, both A03a and B01a have significantly lower spore viability than expected (**Fig 5B**). This in part may be due a genetic load imposed by segregating deleterious alleles. Consistent with our sequencing data, these data indicate that diploidy permits the accumulation of recessive lethal and deleterious mutations on a relatively short time scale.

## Discussion

Whole genome duplications (WGDs) are significant evolutionary events that have profound impacts on genome evolution. Evidence of ancient whole-genome duplication events is found within lineages ancestral to most extant eukaryotic taxa [32–34], including at least two WGDs in the vertebrate lineage [35], and a WGD approximately 100 mya in the *Saccharomyces* lineage [36,37]. In addition, the existence of numerous contemporary polyploid taxa suggests that genome duplication plays a role in short-term adaptive evolution [38]. Genome duplication and polyploidy are also known to increase virulence and aid in stress adaptation in pathogenic fungi [39]. Here, we show that experimental evolution of haploid *Saccharomyces cerevisiae* results in rapid and recurrent WGD. Clones with duplicated genomes arise early in all 46 populations and fix rapidly. We show that concurrent fixation of autodiploids can be attributed to a large fitness effect. Furthermore, the concurrent population dynamics reported here are evidence of a high rate of genome doubling in haploid yeast.

The invasion and subsequent fixation of autodiploids in haploid-founded lineages has been reported before in yeast (see **Table 1**). Some studies report a fitness advantage of WGD in haploid yeast [6], though this is not consistent across studies [16]. Such inconsistency is possibly because the benefit of diploidy is condition-dependent [17]. By employing a competitive growth assay, we demonstrate a relatively large fitness effect of a duplicated genome in our selective environment. A 3.6% fitness effect is substantial: in a recent study we quantified fitness effects of over 116 mutations from 11 evolved lineages in the same conditions, and only 9 conferred a fitness benefit greater than 3.6% [40]. The biological basis of this fitness advantage is unclear. However, there are several strong possibilities. Increased cell size, differential gene regulation, and a diploid-specific proteome [11,41] may all contribute to the adaptive advantage of diploidy. More generally, environmental robustness is often associated with increases in ploidy [38].

The recurrent and remarkably parallel manner in which autodiploids arise and fix points to not only a large fitness effect, but a high rate of occurrence. Our previous work has shown that parallel evolution is evident at the level of genetic pathway and even gene [20,40]. However, the extent of the convergence observed here–where all 46 populations evolve to be autodiploids–is unprecedented in our experimental system. While it cannot be dismissed that some autodiploids were present in the founding inoculum, they are below our 1% detection limit. Autodiploids at this low of a frequency in the inoculum is not sufficient to explain the extent of fixation observed **(S5 Fig).** Simulations indicate the probability of an autodiploid lineage at 1% fixing in 46 out of 46 replicate populations is 2.5 x 10^−3^. Furthermore, given the common dynamics observed in populations of both mating types, autodiploids would have to had arisen in “jackpot” fashion and reach a similar frequency in the inocula of both mating-types. These data strongly support independent WGD events in replicate populations, suggesting a high background rate of duplication. This is consistent with the observation of frequent WGD in mutation accumulation lines ([42], but see conflicting findings using a different strain in [25]). Using a barcode-enrichment assay, Venkataram *et al*. [6] found that roughly half of all evolved clones with increased fitness that arose in a short-term enrichment experiment possessed no mutation apart from a WGD. A recent study found autodiploids to occur in haploid cultures at a rate on the order of 10^−5^ per cell division [26], a rate several orders of magnitude higher than the per base pair mutation rate and sufficiently high to explain repeated autodiploid appearance in this and other haploid-founded evolution experiments.

Given the prevalence of autodiploids in the present evolution experiment, it is worth asking why autodiploids were not reported in a previous haploid evolution experiment in which ostensibly the identical strain and conditions were used [30]. It is possible that in the prior experiment autodiploids did not fix or they could have fixed but were not detected. Despite conscious efforts to maintain identical selective environments, subtle differences in the conditions may exist given that evolution experiments were conducted years apart in different facilities. Indeed, inconsistency in the appearance of WGD across experiments and conditions is common in the field [5,15]. Even subtle differences in the evolution conditions could shift the selective benefit of autodiploidy and yield population dynamics different from those seen here. Alternatively, it is possible that autodiploids did fix in the previous haploid evolution experiment but went undetected. The populations analyzed in the haploid study were part of a larger ~600 population experiment, and the 40 focal populations were selected based on the presence of a sterile phenotype. Mutations producing sterile phenotypes are predominantly adaptive and recessive loss-of-function [43]. The presence of such beneficial mutations would have biased the selection of populations towards those retaining haploidy. We analyzed a subset of the remaining ~560 populations by DNA content staining and find that ~30% (3 of 10) of them appear autodiploid at generation 1,000, though this is still less frequent than we report here. Further at least one of the forty sequenced populations (RMS1-E09, 30) which appeared to be an autodiploid based on the presence of a large number of mutations present at a frequency of 0.5, was confirmed as 2N through ploidy-staining.

The consequences of WGD are apparent on both the phenotypic and genotypic level. One such consequence is the susceptibility of autodiploids to Haldane’s sieve, resulting in a “depleted” spectrum of beneficial mutations. We find a decline in adaptation rate following WGD, which mirrors findings from studies that directly compare the rates of haploid population adaptation with that of diploids [20,27]. This implies a fitness tradeoff in the shift from 1N to 2N, wherein the fixation of a large-effect beneficial genotype comes at the cost of eliminating access to future recessive beneficial mutations. This tradeoff associated with genome duplication is predicted when population size is large and most beneficial mutations are partially or fully recessive [44], conditions that are met in our populations [20,30].

Autodiploids share physiological traits with both haploid and diploid cell types. Like their haploid founders, autodiploids possess only a single mating-type allele and will readily mate with cells of the opposite mating-type, indicating haploid-specific regulation of mating-pathway genes. As with diploids, autodiploids possess a 2N genome and exhibit larger cell size [11]. Consequently, we observe some overlap in the spectrum of beneficial mutations. We have identified targets of selection shared between haploids and autodiploids along with targets specific to autodiploids. While several targets were mutual to haploids and autodiploids, the extent of recurrence varied by gene. For example, *IRA1* mutations were common to both ploidies but enriched in haploids. In contrast, there were five ploidy-specific genes that were targets in autodiploids but never mutated in haploids. These genes (*PHO81*, *YTA7*, *PHO4*, *IRC8*, and *PSA1*) represent targets of selection that are specifically enriched in autodiploids, suggesting that WGD may expose adaptive pathways that are not easily accessible to either haploids or diploids. The functional basis of selection on a few common genic targets reported here has been investigated [45,46], and many targets have been observed in evolution experiments before (**S1 Table**). However, little is known about the functional consequences of most mutations identified here.

Genome duplication also has consequences on genome stability and the evolution of structural variation. Across our 46 populations we identify 6 independently evolved aneuploidies and 20 independently evolved structural variants. Structural variants are more frequent in autodiploid genomes than in evolved haploid genomes of the same background, even after accounting for length of evolution. Haploids are constrained: whereas the structural variants observed in haploids always result in a net gain of genetic material, autodiploid structural variants include both amplifications and deletions. The ability to generate a greater degree of structural variation could provide a secondary advantage to WGD. Aneuploidies, large rearrangements, and CNVs have been shown to arise and confer an advantage in experimentally evolving yeast populations [47,48]. Of note, several of the recurrent structural arrangements described in the present study, including trisomy III and a 317 kb deletion on Chr. III, have previously been described as beneficial [49]. The observation of both gain and loss of genetic material from Chr. III may indicate complex selection on phenotypes unachievable through point mutations.

Loss of heterozygosity (LOH) provides a means of overcoming the masking effect of ploidy in autodiploids allowing recessive beneficial mutations to become homozygous. Analysis of the distribution of homozygous mutations across evolved autodiploid genomes reveals LOH frequently occurs in two locations: on the right arm of Chr. XII and the right arm of Chr. XV. The right arm of Chr. XII has been characterized as a hotspot for LOH in experimental and natural populations [20,50] mediated by a high rate of recombination at the rDNA repeats [51]. To our knowledge, a mitotic recombination hotspot on Chr. XV has not been described. Recurrent LOH may have substantial evolutionary implications as the affected regions may experience different rates of genome evolution and divergence than the rest of the genome. On the one hand, fitness may decline dramatically due to the exposure of deleterious mutations to selection. On the other hand, the rate of adaptation may be increased by providing access to recessive beneficial mutations that would otherwise be masked by Haldane’s sieve. Theory predicts that sufficient mitotic recombination may allow asexual populations to circumvent Haldane’s sieve [52]. While we only show prevalence of LOH and not functional evidence of adaptive LOH, such events have been repeatedly observed in adapting yeast populations [53,54]. Further, the LOH on Chr. XV was not detected previously in diploids [20], an observation that is more easily explained by selection than a change in the rate of occurrence.

The same masking effect that stifles recessive beneficial mutations is also predicted to permit the accumulation of deleterious mutations in diploids [19]. In evolved haploid populations few if any deleterious mutations fix: previously only 1 of 116 evolved mutations was characterized as putatively deleterious [40]. We show that, in contrast to haploid genomes, evolved autodiploid genomes harbor an abundance of putative recessive lethal mutations (**Fig 5A**). We sporulated autodiploids with normal 2N karyotypes by complementing the *MAT*α information on a plasmid. We find evidence of the accumulation of both lethal and deleterious mutations as indicated by a large number of inviable and slow-growing haploid spores (**Fig 5B**). Autodiploids are initially buffered from the effects of de novo recessive deleterious alleles due to the presence of a second, functional allele. With each successive heterozygous recessive deleterious mutation that fixes, the reduction of functional ohnologs to one eliminates genetic redundancy. Loss of redundancy shifts the distribution of fitness effects (DFE) and an increase in the target size for lethal or deleterious mutations. Over evolutionary time the collective shift in the DFE would impact rate of adaptation.

Interestingly, loss of redundancy occurred rapidly following the historical yeast WGD [55]. Here we show that recessive deleterious and lethal mutations can accumulate shortly after WGD. On a population level, the increased target size for mutations as well as the masking of deleterious mutations may increase standing variation between selective sweeps and may explain populations with deeply diverging clones (**S8 Fig**).

Whole genome duplications occur via autoduplication, wherein the two genomes arise from the same species, or alloduplication, wherein two divergent genomes are brought together through a hybridization event [56]. The WGD events observed here are autoduplications analogous to the origin of autopolyploid taxa [57] and to endoreplication events in somatic eukaryotic cells [58]. The patterns reported here nonetheless inform our understanding of post WGD adaptation. The ancient WGD in the *Saccharomyces* lineage is thought to have occurred by alloduplication followed by LOH at the mating-type locus to restore fertility [59,60], and therefore would have gone through an intermediate asexual ‘duplicated’ diploid state, similar to the *MAT***a**/**a** and *MAT*α/α populations investigated here. We demonstrate that this cell type has a direct fitness advantage over an isogenic haploid cell type. The immediate fitness gain of WGD is accompanied by several evolutionary tradeoffs that impact future adaptability including a reduced rate of adaptation, shifted distribution of beneficial mutations, karyotype changes, and the accumulation of recessive deleterious and lethal mutations that reduce redundancy in the duplicated genome.

## Methods

### Strain construction

*MAT***a**/**a** strains were constructed for fitness assays by converting yGIL701, a fluorescently labeled *MAT***a**/α diploid isogenic to our ancestral haploid background, to *MAT***a**/**a**. yGIL701 was struck out and 10 separate clones were selected. Clones were transformed with pGIL088, which encodes a gal-inducible *HO* and a *MAT***a** specific *HIS3* marker. 5 ml cultures of YPD were inoculated with a single transformant for each starting clone. Cultures were grown for 48 hours, allowing for glucose to be depleted and catabolite repression of *GAL* genes to be lifted. After 48 hours 100 μl of each culture was plated to SD–his. Histidine prototrophs were screened in α-Factor (Sigma) for shmoos. Confirmed strains were used in competition assays.

### Evolution experiment

Experimental populations were founded with 130 μl of isogenic W303 ancestral culture; 22 with yGIL432 (*MAT***a**, *ade2-1*, *CAN1*, *his3-11*, *leu2-3*,*112*, *trp1-1*, *URA3*, *bar1*Δ::*ADE2*, *hm*lαΔ::*LEU2*, *GPA1*::NatMX, *ura3*Δ::P*FUS1*-yEVenus), and 24 with yGIL646, a *MAT*α strain otherwise isogenic to yGIL432 (**S11 Fig**). Populations analyzed here were evolved in separate wells of a 96-well plate. Ancestral strains were grown as 5 ml overnight cultures from single colonies prior to 96 well plate inoculation. This founding plate was propagated forward and then immediately frozen down.

All populations analyzed here were evolved in rich glucose (YPD) medium. Cultures were grown in unshaken 96-well plates at 30°C and were propagated every 24 hours via serial dilutions of 1:1024. Approximately every 60 generations, populations were cryogenically archived in 15% glycerol.

### Fitness assays

Fitness assays were performed as described previously [40]. Evolved autodiploid populations were mixed 1:1 with a version of the ancestral strain (yGIL432 or yGIL646, genotypes listed above) labeled with ymCitrine at *URA3*. Cultures were propagated in a 96-well plate in an identical fashion to the evolution experiment for 40 generations. Every 10 generations, saturated cultures were sampled for flow cytometry. Analysis of flow cytometry data was done using FlowJo 10.3. Selective coefficient was calculated as the slope of the change in the natural log ratio between query and reference strains. Assays were performed for all 46 evolved populations at 16 time points between generations 0 and 4,000.

To measure the fitness effect of autodiploidy, fitness assays were performed as described above, using instead a non-labeled version of yGIL432 as a reference. This strain was mixed 1:1 with either a fluorescently-labeled version of the same strain or one of ten biological replicate fluorescently labeled diploid strains. The fitness of each autodiploid reconstruction was calculated as the mean fitness across 12 replicate competitions.

Adaptation rates for each autodiploidized lineage were calculated as the rate of change in relative fitness between generation 0 and the time point at which diploids were present at over 98%. For comparison, rate of adaptation was also calculated for diploid-founded populations evolved in parallel [20]. The median time point of autodiploid fixation was generation 600 for the haploid-founded dataset. To generate a comparable dataset, rates of adaptation for diploids were calculated from generations 0–600 and 600–4000. Rates were compared in SPSS using a repeated measures ANOVA with two within subject factors (time) and two between subject factors (haploid-founded and diploid-founded). Because some groups violated homogeneity assumptions, post-hoc analysis was done using a Bonferroni correction.

### DNA content analysis

Focal populations for DNA content analysis were objectively chosen by randomly selecting one 8-well column per mating-type from the 96-well plate. Time-course ploidy states of 16 focal evolved populations were assayed through flow cytometry analysis of DNA content as described in Gerstein and Otto [16]. Briefly, 10 μl of each sample were inoculated in 3 ml YPD and grown overnight. 100 μl of saturated cultures were then diluted 1:50 into YPD and grown to mid-log. To arrest in G1, 1 ml mid-log culture was transferred into 200 μl 1M hydroxyurea and incubated on a 30°C roller drum for 3 hours. Cultures were then fixed with 70% ethanol, treated with RNAse and proteinase K, stained with Cytox green (Molecular Probes), and analyzed on a BD FACSCanto. Haploid and diploid frequencies were estimated using FlowJo v10.3 by fitting data to Watson-Pragmatic cell cycle models. This method of estimation was validated with a series of known ploidy mixtures (**S9 Fig**).

### Simulations

Simulations of lineage trajectories were performed using a forward-time algorithm designed to imitate the conditions in the evolution experiment reported here. Simulation code, which is described in [61], was provided by E. M. Frenkel and can be accessed at https://github.com/genya/asexual-lineage-adaptation. Estimates for the distribution of fitness effects (an exponential distribution with mean $\bar{s}$ = 0.85%) and beneficial mutation rate (*U*_*b*_ = 1.0 x 10^−4^) were kept as described previously [61]. This model assumes the spectrum of mutations available to haploids is the same as the spectrum available to autodiploids. Simulations were performed with constant inputs for DFE parameters, beneficial mutation rate, inoculation time of the focal lineage (generation *t* = 0), and fitness advantage of the focal lineage (*s*_*0*_ = 3.6%). The initial frequency of the focal lineage was varied (*f*_*0*_ = 0.01%-1.0%) for each set of simulations, and a total of ten thousand simulations were performed for each *f*_*0*_.

### Sequencing

Evolved clones were obtained by streaking evolved populations to singles on YPD and selecting two clones per population. These clones were grown to saturation in 5 ml YPD and then spun down to cell pellets and frozen at -20°C. Genomic DNA was harvested from frozen pellets via phenol-chloroform extraction and precipitated in ethanol. Total genomic DNA was used in a Nextera library preparation. The Nextera protocol was followed as described previously [40]. All individually barcoded clones were pooled and sequenced on 2 lanes of an Illumina HiSeq 2500 sequencer by the Sequencing Core Facility at the Lewis-Sigler Institute for Integrative Genomics at Princeton.

### Sequencing analysis

Two lanes of raw sequence data were concatenated and then demultiplexed using a custom python script (barcodesplitter.py) from L. Parsons (Princeton University). Adapter sequences were trimmed using the fastx_clipper from the FASTX Toolkit. Trimmed reads were aligned to an S288c reference genome version R64-2-1 [62] using BWA v0.7.12 [63] and variants were called using FreeBayes v0.9.21-24-381 g840b412 [64]. Roughly 10,000 polymorphisms were detected between our ancestral W303 background and the S288c reference, and the corresponding genomic positions were removed from analysis. All remaining calls were confirmed manually by viewing BAM files in IGV [65]. Zygosity was determined based on read depth and allele frequency (**S2B Fig**). Mutations were classified as fixed if present in all clones from a population. Clones were genotyped for *MAT* alleles by identifying mating-type specific sequences within the demultiplexed FASTQ files. Ancestral polymorphisms were inferred using VCFTools [66] to identify homozygosities shared by all clones of the same mating-type. Six mating-type specific SNPs were removed from downstream analysis following verification of homozygosity.

Clone genomes were each independently queried for structural variants. Following BWA alignment, coverage at each position across the genome was calculated. Aneuploidies were detected by calculating median chromosome coverage and dividing this by median genome-wide coverage for each chromosome, producing an approximate chromosome copy number relative to the duplicated genome (**Fig 4; S2 Dataset**). CNVs were detected by visual inspection of chromosome coverage plots created in R (**S10 Fig; S3 Dataset**).

### Phylogenetic analysis

Variants identified by SNPeff were used to infer a phylogeny based on 7,932 sites containing 4,742 variable sites, either SNPs or small indels (**S8 Fig**). Evolved and ancestral sequences (n = 93) were aligned with MUSCLE. A general time reversible substitution model with uniform rates (-lnL = 44803.45) was selected based on jModelTest. A maximum likelihood tree was then constructed and rooted by the ancestor in MEGA. Subclades were found to be due to incomplete linage sorting of mitochondrial polymorphisms. After phylogenetic analysis it was evident that four clones were originally attributed to incorrect populations. Tight clustering and short branch lengths suggests either very recent contamination or an issue during colony isolation (populations were struck out two to a plate on bisected YPD plates). In the text, these clones are identified by the suffix “c” and are attributed to the population to which they are most phylogenetically similar.

### Identification of common targets and ploidy-enriched targets

A recurrence approach was utilized to identify common targets of selection. A random distribution of the 3,431 coding sequence (CDS) mutations across all 5,800 genes predicts only two genes to be mutated more than five times by chance alone. We determined the probability that chance alone explains the observed number of mutations of each gene by assuming a random distribution of the 3,431 mutations across the 8,527,393 bp genome-wide CDS. Common targets of selection were defined as genes with five or more CDS mutations and a corresponding probability of less than 0.1% (**Fig 2A**). Notably, analysis using only nonsynonymous mutations identified largely the same set of common targets of selection as did analysis using all CDS mutations. To determine which targets of selection are impacted by ploidy, our recurrence approach was used to analyze mutations in a previously published *MAT***a** haploid dataset (**S7 Fig)** [30,40]. We compared the probability of the observed number of CDS mutations in each gene between ploidies (**Fig 2C**). A gene was considered ploidy-enriched if the ratio of probabilities was at least 10^5^.

### Evolved clone sporulation and tetrad dissection

Three clones (A02a, B01a, C03b) for which genome sequence data revealed no aneuploidies were selected for sporulation. Evolved *MAT***a**/**a** clones were transformed with pGIL071 which encodes the α2 gene necessary for sporulation and a *URA3* marker for selection. Transformants were sporulated in Spo++ -ura media. Following 72 hours, sporulation efficiency was calculated via hemocytometer, cultures were digested with zymolyase, and tetrads were dissected on YPD agar plates. Spores were incubated 48 hours and then assayed for germination. Control strain yGIL1039, made by crossing yGIL432 to yGIL646 and converting the resulting diploid to *MAT***a/a** as described above, was transformed and dissected in parallel.

### Data deposition

The short-read sequencing data reported in this paper have been deposited in the NCBI BioProject database (accession no. PRJNA422100).

## Supporting information

S1 Fig Distribution of evolved mutations across the genome. Shown are the genomic positions of all 5,061 nuclear mutations. Mutations are colored by type: nonsynonymous-yellow, synonymous-green, intergenic-blue, tRNA-magenta.
(EPS)

S2 Fig Distribution of autodiploid mutations and criteria for zygosity determination. A) The number of total mutations per clone follows a normal distribution. B) Total calls (reference + alternate) and mutation frequency for each of the 8,305 nuclear mutations across the 92 autodiploid clones. Dotted lines define the criteria for zygosity. Mutations are colored by zygosity: homozygous-red, heterozygous-blue, inconclusive-green.
(EPS)

S3 Fig Autodiploids sweep through haploid-founded populations. Autodiploids were tracked in 16 focal popula- tions via time-course DNA content staining. Autodiploid lineages arise quickly in all 16 populations and fix by genera- tion 1,000 in all but 2 populations. *MAT***a** (*n* = 8) and *MATα* (*n* = 8) are represented by red and blue lines, respectively.
(EPS)

S4 Fig Individual traces for fitness and ploidy for 13 populations. Time-course ploidy (red) and fitness (orange) dynamics across 4,000 generations for the 13 populations for which both have been measured.
(EPS)

S5 Fig Simulations of autodiploid fixation. A) The probability of autodiploid fixation based on simulations at starting frequencies ranging from 0.0001 to 0.01. Each data point represents the proportion of populations that fix autodiploids in 10,000 simulations. B) Heatmap showing the probability distributions of autodiploid fixation at a given starting frequency.
(EPS)

S6 Fig Identification of overrepresented tRNA genes in autodiploids. Plotted on the x-axis is the observed number of mutations in each of the 300 tRNA genes in the S288c reference genome. On the y-axis is the probability that the observed number of mutations in each tRNA gene occurred by chance. The only common tRNA target of selection (red circle) occurred independently five times and is below the probability threshold of 0.1% that was set by the recurrence model for protein coding mutations.
(EPS)

S7 Fig Identification of common targets of selection in haploids. Plotted on the x-axis is the observed number of coding sequence (CDS) mutations in each of the 5800 genes in the S288c reference genome. On the y-axis is the probability that the observed number of CDS mutations in each genes occurred by chance. Common targets of selection are genes with 3 or more CDS mutations and corresponding probability of less than 0.1%. Common targets of selection are shown as red circles and labeled by gene name. Sequencing data for haploids were reported previously (Lang *et al* 2013).
(EPS)

S8 Fig Phylogenetic tree of evolved autodiploid clones. A maximum likelihood phylogeny based on 4,742 variable sites.
(EPS)

S9 Fig Validation of hydroxyurea-arrest assay. The hydroxyurea (HU) arrest assay and data analysis approach was validated by preforming FACS analysis on prefixed control cultures. Measures for ploidy frequency using the assay and analysis were largely accurate when compared to actual measured frequencies.
(EPS)

S10 Fig Identification of copy number variants by sequencing coverage across chromosomes. Each coverage map corresponds to a chromosome that contains the identified copy number variant (CNV) listed above. For reference, a control autodiploid clone (with no CNV’s) is shown above. Maps are labeled by clone/population. The x-axis is chromosome position, scaled to length. The y-axis is coverage, scaled to median genome-wide coverage. Red dashed boxes highlight the detected CNV.
(EPS)

S11 Fig Ancestral strain construction. All strains are identified by their number in the Lang Lab yeast collection (yGIL prefix). Both the MAT**a** (yGIL432) and MATα (yGIL646) founders were derived from a common diploid (yGIL103) and engineered to contain the BY allele of *GPA1* and *yEVenus* fluorescent marker at the *URA3* locus. Circles indicate backcrosses to the strains listed above. Shown are the six polymorphic sites (gene and amino acid change) that differ between the founders.
(EPS)

S1 Dataset All 8,305 de novo mutations detected across the 46 autodiploid populations. (XLSX)

S2 Dataset Aneuploidies detected by sequencing read depth. (XLSX)

S3 Dataset Copy number variants detected by sequencing read depths. (XLSX)

S1 Table (DOCX)
