# Supplementary material for: Adaptive genome duplication affects patterns of molecular evolution in Saccharomyces cerevisiae
Source: PLoS Genet. 2018 May 25;14(5):e1007396. doi: 10.1371/journal.pgen.1007396 (PMC5991770; doi:10.1371/journal.pgen.1007396)
Supplement: S1 Table — (DOCX) [file pgen.1007396.s015.docx]

| **Gene** | **Number of Mutations (Populations)** | | | **Evolved Alleles** | **GO Biological Process Term**^3^ |
| --- | --- | --- | --- | --- | --- |
|  | **Homozygous** | **Heterozygous** | **Mixed**^2^ |  |  |
| *KRE6* | 1 | 19 | 1 | T311R, A351T, D361H, T376N, R380G, S393L, W447L, W447L, C454F, S464Y, P487S, G492D, E497D, D499A, S517F, N545K, Y579*, G590D, W642C, D654G, Q681* | Fungal-type cell wall organization |
| *CCW12* | 1 | 14 | 0 | M1Startloss, S39del, C40F, E41*, S50C, D62Y, Q67*, Q67*, Y68fs, T70N, E77*, E93*, Y110*, L125S, G127S | Fungal-type cell wall organization |
| *PTR2* | 2 | 12 | 0 | A43A, G110fs, G128R, M203T, V243F, C279F, W313S, P359R, A391P, Y452fs, S484Y, A491G, K500E, Y555* | Peptide transport |
| *IRA1* | 2^1^ | 11 | 0 | L37F T39fs N137K, Q550*, S622*, T820M, L974*, I1437I, F1489F, S1603G, S1753I, C1754fs, C2067* | Negative regulation of Ras protein signal  transduction |
| *PSE1* | 0 | 12 | 0 | L107*, W137S, Q308fs, W331*, L372V, I517L W606*, V697fs , Q739*, E765Q, L869*, S1006* | Protein import into nucleus |
| *WHI2* | 6^1^ | 3 | 2^1^ | Q29*, S72*, L76fs, L76fs, Q81*, E168G, Q181*, N275fs, T283fs, A310P, A338* | Regulation of growth |
| *LTE1* | 0 | 11 | 0 | S185*, W380*, E653*, A748S, A748V, E865*, Q916K, M1062I, K1138*, A1368fs, W1403* | Regulation of exit from mitosis |
| *YTA7* | 0 | 9 | 1 | S319S, E475*, P564H , P675Q, L803F, L965F, I1032S, P1061R, R1120fs, A1203P | Negative regulation of transcription |
| *PHO81* | 4^1^ | 6 | 0 | R93*, F96L, N244N, N329fs, A582fs, E621*, P699Q, L748fs, PR753*, V1079fs | Phosphate-containing compound metabolic  process |
| *ACE2* | 2 | 7 | 1 | E213*, R227H, E235*, P288Q, S299*, P314fs, N324fs, S473*, S694*, L770fs | Positive regulation of cell separation after  cytokinesis |
| *PDR5* | 2 | 7 | 0 | T39I, Q56L, S197F, A262S, D1035D, N1120K, F1224Y, V1290V, S1331Y | Drug transport |
| *SFL1* | 3^1^ | 5 | 0 | E4E, G88R S114R, S213*, S283fs, P432H, DY544*, Y545* | Negative regulation of invasive growth |
| *SIM1* | 0 | 8 | 0 | A119fs, L132L, G222G, G234C, V235G, P344L, L418W, A427T | Fungal-type cell wall organization |
| *IRC8* | 2^1^ | 5 | 0 | L262*, Q274*, L310L, N316fs, D474fs, Q629E, L649* | Mitotic recombination |
| *LCB2* | 0 | 7 | 0 | H44H, F148fs, T149I, G373C S414N, R494T, S526* | Sphingolipid biosynthetic process |
| *ANP1* | 0 | 7 | 0 | K2*, R82fs, S120C, P195Q, V230L, IQ241*, G303W | Protein N-linked glycosylation |
| *CTS1* | 5^1^ | 0 | 0 | L69*, L83fs, C96Y, Q234*, E298* | Cell separation after cytokinesis |
| *PSA1* | 0 | 5 | 0 | R15K, P24A, G284S, L293S, D330T | Cell wall mannoprotein biosynthetic process |
| *PHO4* | 0 | 5 | 0 | F171F, D175fs, L270V, V286M, A298fs | Positive regulation of phosphate metabolic process |
| *STE4* | 0 | 5 | 0 | G250G, S261fs, A287S, R312Q, E315*, Q337* | Pheromone-dependent signal transduction |

^1^Includes mutations putatively homozygous with inconclusive coverage.

^2^Mixed mutations are present as both homozygotes and heterozygotes in the population.

^3^GO terms were manually curated using the Yeast Genome Database (yeastgenome.org).
